# Polyphenol Containing Sorghum Brans Exhibit an Anti-Cancer Effect in Apc Min/+ Mice Treated with Dextran Sodium Sulfate

**DOI:** 10.3390/ijms22158286

**Published:** 2021-08-01

**Authors:** Seong-Ho Lee, Hee-Seop Lee, Jihye Lee, Darshika Amarakoon, Zhiyuan Lou, Leela E. Noronha, Thomas J. Herald, Ramasamy Perumal, Dmitriy Smolensky

**Affiliations:** 1 Department of Nutrition and Food Science, College of Agriculture and Natural Resources, University of Maryland, College Park, MD 20742, USA; hslee123@umd.edu (H.-S.L.); jlee1232@terpmail.umd.edu (J.L.); darshika@umd.edu (D.A.); zhiyuanlou@hotmail.com (Z.L.); 2 Arthropod Borne Animal Disease Research Unit, Agricultural Research Service, U.S. Department of Agriculture, Manhattan, KS 66502, USA; Leela.noronha@usda.gov; 3 Grain Quality and Structure Research Unit, Agricultural Research Service, U.S. Department of Agriculture, Manhattan, KS 66502, USA; thomasherald98@gmail.com; 4 Agricultural Research Center, Kansas State University, Hays, KS 67601, USA; perumal@ksu.edu

**Keywords:** high phenolic sorghum bran, colon cancer, PI3K, AMPK, autophagy, Apc Min/+ mouse

## Abstract

Colon cancer (CC) is considered a high-risk cancer in developed countries. Its etiology is correlated with a high consumption of red meat and low consumption of plant-based foods, including whole grains. Sorghum bran is rich in polyphenols. This study aimed to determine whether different high-phenolic sorghum brans suppress tumor formation in a genetic CC rodent model and elucidate mechanisms. Tissue culture experiments used colorectal cancer cell lines SW480, HCT-116 and Caco-2 and measured protein expression, and protein activity. The animal model used in this study was APC Min+/mouse model combined with dextram sodium sulfate. High phenolic sorghum bran extract treatment resulted in the inhibition of proliferation and induced apoptosis in CC cell lines. Treatment with high phenolic sorghum bran extracts repressed TNF-α-stimulated NF-κB transactivation and IGF-1-stimulated PI3K/AKT pathway via the downregulation of β-catenin transactivation. Furthermore, high-phenolic sorghum bran extracts activated AMPK and autophagy. Feeding with high-phenolic sorghum bran for 6 weeks significantly suppressed tumor formation in an APC Min/+ dextran sodium sulfate promoted CC mouse model. Our data demonstrates the potential application of high-phenolic sorghum bran as a functional food for the prevention of CC.

## 1. Introduction

Sorghum (*Sorghum bicolor* (L.) Moench) is the fifth most cultivated cereal crop destined for human consumption worldwide. Sorghum is a high-energy and drought-resilient crop because of the higher conversion of both solar energy and water use [1]. In addition, owing to its ability to adapt, sorghum is an important crop in the era of climate change [2]. Sorghum is grown in most of the states in the United States; however, the majority of sorghum production comes from Kansas, Texas, Oklahoma and Colorado. As a food product, it represents a profitable market for farmers and an interesting topic for research into human consumption. Grain sorghum is of great importance to American growers, especially in the Great Plains area, because of its feed value and abundance of major nutrients (starch, proteins, lipids, minerals and vitamins) [3]. Indeed, some species with pharmaceutical value contain diverse phytonutrients in the form of phenolic compounds, including phenolic acids (benzoic and cinnamic acids), flavonoids (anthocyanidins) and tannins (proanthocyanidins, flavin-3-ols), which are mostly concentrated in the bran portion [4].

Several in vitro studies have reported on the strong antioxidative and anti-inflammatory capacity of sorghum compounds [5,6]. Moreover, sorghum consumption may be beneficial for the prevention of cancer. Yang et al. previously reported that sorghum extract has an inhibitory effect on HT-29 colon cancer cells [7]. In addition, sorghum bran may modulate colitis [8,9] and inhibit chemically induced colon cancer formation in rats treated with azoxymethane [10]. The intragastric administration of *Hwanggeumchal* sorghum extract has been shown to reduce metastasis of breast cancer cell lines in immune-deficient mice [11]. We previously demonstrated that sorghum extract high in phenols can inhibit cancer cell growth through specific mechanisms [12,13,14].

Some sorghum accessions have a higher total phenolic content and more antioxidant compounds than those in other food sources [15,16]. However, the level of phenolic compounds is affected by several factors, including the sorghum genotype, environmental stress, and processing and extraction methods [17]. While such findings are of great value to the American public and to the sorghum community, there remains a lack of information regarding mechanisms and a failure to identify a sorghum genotype(s) with a high amount of relevant bioactive compounds for cultivation in the United States. Recently, we identified a novel high phenolic sorghum bran extract (HP; accession number PI570481) [12] which showed a significant suppression of cell proliferation and induction of apoptosis in human colon cancer cells, which were associated with the up- and downregulation of cell cycle and apoptosis-regulating genes [14].

The aim of the present study was to elucidate the biochemical and molecular anti-cancer mechanisms of high phenolic sorghum using human colon cancer cells and examine whether consumption of high phenolic sorghum represses the formation and development of tumors using a genetic colon cancer rodent model.

## 2. Results

### 2.1. High Phenolic Sorghum Bran Extracts Repress Proliferation and Induce Apoptosis in Human Colon Cancer Cells

**Figure 1:**
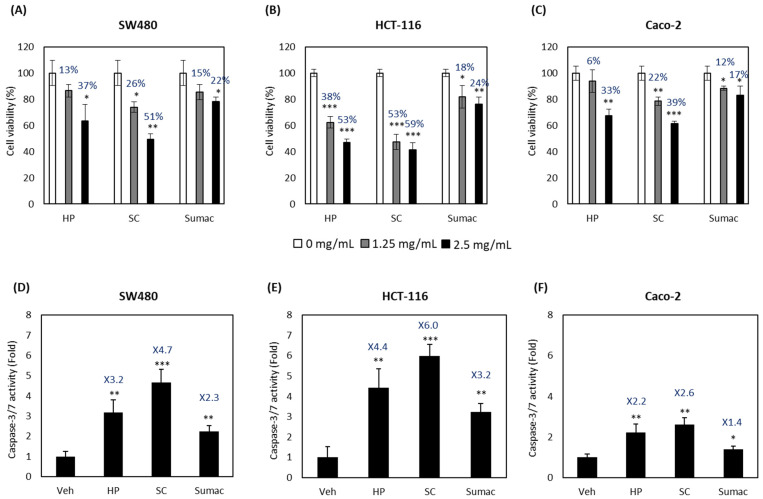
High phenolic sorghum bran extracts repress the proliferation and induce the apoptosis of human colon cancer cells. (**A**–**C**) Three human colon cancer cells, SW480 (**A**), HCT-116 (**B**) and Caco-2 (**C**) cells were treated with 0, 1.25 and 2.5 mg/mL of sorghum bran extracts (HP, SC and Sumac) for 24 h and cell proliferation was measured using MTT assay. (**D**–**F**) SW480 (**D**), HCT-116 (**E**), and Caco-2 (**F**) cells were treated with 2.5 mg/mL of sorghum bran extracts (HP, SC and Sumac) for 24 h and apoptosis was measured using caspase 3/7 enzyme assay kit (Promega). Values are the mean ± SD (*n* = 3). Significant differences between the vehicle- and sorghum bran extract-treated groups are indicated as * *p* < 0.05, ** *p* < 0.01, *** *p* < 0.001.

We compared the anti-proliferative and pro-apoptotic activities of HP with two other sorghum bran extracts (SC and Sumac) using human colon cancer cell lines, including two invasive (metastatic) cell lines (SW480 and HCT-116) and a non-invasive cell line (Caco-2). As shown in Figure 1A–C, HP, SC and Sumac significantly inhibited the growth of all three human colon cancer cells in a dose-dependent manner. We also measured the apoptotic activity of sorghum bran extracts at 2.5 mg/mL by measuring the caspase 3/7 enzyme activity. The apoptotic activity was increased in all three colon cancer cell lines, and fold induction was much higher in invasive cancer cell lines (SW480 and HCT-116) than in non-invasive Caco-2 cells (Figure 1D–F).

### 2.2. High-Phenolic Sorghum Bran Extracts Modulate NF-κB Activity in Human Colon Cancer Cells

**Figure 2:**
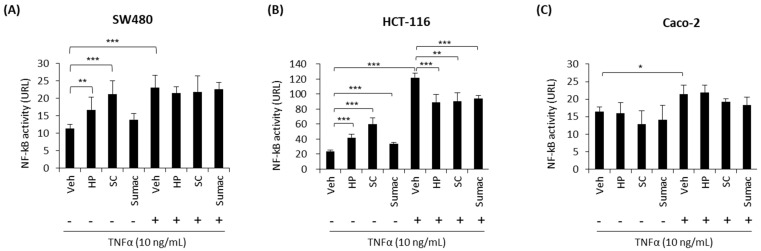
High phenolic sorghum bran extracts modulate NF-κB activity in human colon cancer cells. SW480 (**A**), HCT-116 (**B**) and Caco-2 (**C**) cells were transfected with *NF-kB* and *pRL null* luciferase plasmid for 24 h and pretreated with 2.5 mg/mL of sorghum bran extracts for 1 h and additionally incubated in the absence or presence of TNF-α (10 ng/mL) for 6 h. Luciferase was assayed using Dual Luciferase Assay System (Promega). Values are the mean ± SD (SW480, *n* = 6; HCT-116, *n* = 4; Caco-2, *n* = 3). Significant differences between two groups are indicated as * *p* < 0.05, ** *p* < 0.01, *** *p* < 0.001.

NF-κB signaling is a key regulator of colon cancer hallmarks, including colitis, proliferation, and apoptosis. An increased activation of the NF-κB pathway is one of the features of colon cancer development, and the suppression of the NF-κB signaling pathway using nutritional and pharmacological inhibitors is an effective therapeutic approach for the treatment of colon cancer [18]. To determine whether sorghum bran extracts (HP, SC, and Sumac) inhibit the TNF-α-stimulated NF-κB pathway, we transfected NF-κB luciferase reporter genes into human colon cancer cells and treated with 2.5 mg/mL of sorghum extracts in the presence or absence of TNF-α. The results indicate that HP and SC significantly increased the transcriptional activity of NF-κB in SW480 and HCT-116 cells (Figure 2A–C) in the absence of a stimulator (TNF-α). However, HP, SC and Sumac suppressed TNF-α-stimulated NF-κB transcriptional activity in HCT-116 cells, but not in SW480 and Caco-2 cells. In addition, the response to TNF-α was much weaker in Caco-2 cells than in SW480 and HCT-116 cells (Figure 2A–C).

### 2.3. High Phenolic Sorghum Bran Extracts Downregulate the Transcriptional Activity of β-Catenin and Its Expression in Human Colon Cancer Cells

**Figure 3:**
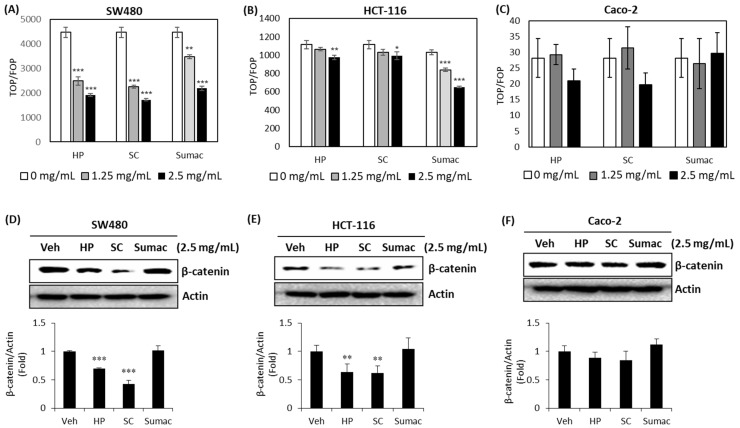
High phenolic sorghum bran extracts downregulate β-catenin expression and its transcriptional activity in human colon cancer cells. (**A**–**C**) Three human colon cancer cells, SW480 (**A**), HCT-116 (**B**) and Caco-2 (**C**) cells were transfected with *TOP* or *FOP* with *pRL null* luciferase plasmid for 48 h and then treated with 0, 1.25 and 2.5 mg/mL of sorghum bran extracts for 24 h. Luciferase was assayed using Dual Luciferase Assay System (Promega). (**D**–**F**) SW480 (**D**), HCT-116 (**E**) and Caco-2 (**F**) cells were treated with 2.5 mg/mL of sorghum bran extracts (HP, SC and Sumac) for 24 h and Western blotting was performed for β-catenin and actin. Values are the mean ± SD (*n* = 3). Significant differences between vehicle- and sorghum bran extract-treated groups are indicated as * *p* < 0.05, ** *p* < 0.01, *** *p* < 0.001.

Adenomatous polyposis coli (APC) mutation is the most common (90%) genetic alteration in colon cancer patients and its downstream target, β-catenin, is highly associated with cancer cell survival and proliferation. Thus, the targeting of β-catenin using natural products is a promising strategy for cancer prevention and therapy [19]. The *TOP/FOP* flush reporter gene can be used to measure transcriptional activity and is a good indicator of the downstream effects of β-catenin. To determine whether high phenolic sorghum extracts affect the transcriptional activity of β-catenin, we transfected *TOP* and *FOP* flush luciferase reporter genes into human colon cancer cells, treated them with sorghum bran extracts, and measured the resulting luciferase activity. All three sorghum bran extracts were found to repress the transcriptional activity of β-catenin significantly in a dose-dependent manner in SW480 and HCT-116 cells, but not in Caco-2 cells (Figure 3A–C). Since the transcriptional activity of β-catenin is associated with several mechanisms, including its expression and/or stability, we measured β-catenin expression. As shown in Figure 3D–F, HP and SC reduced the expression of β-catenin in SW480 and HCT-116 cells, but not in Caco-2 cells.

### 2.4. High Phenolic Sorghum Bran Extracts Weaken the IGF-1 (Insulin-Like Growth Factor-1)-Stimulated PI3K/AKT Pathway in Human Colon Cancer Cells

**Figure 4:**
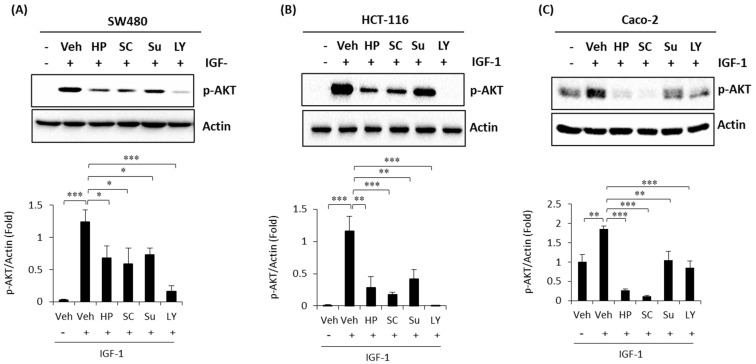
High phenolic sorghum bran extracts weaken the IGF-1-stimulated PI3K/AKT pathway in human colon cancer cells. SW480 (**A**), HCT-116 (**B**) and Caco-2 (**C**) cells were pretreated with sorghum bran extracts (HP, SC, Sumac; 2.5 mg/mL) and LY294002 (selective inhibitor of PI3K; 50 µM) for 6 h and then co-treated with IGF-1 (100 ng/mL) for 2 h. Western blot was performed for phospho-AKT and actin. Su, Sumac; LY, LY294002 (selective inhibitor of PI3K). Values are the mean ± SD (*n* = 3). Significant differences between two groups are indicated as * *p* < 0.05, ** *p* < 0.01, *** *p* < 0.001.

The PI3K (Phosphoinositide 3-kinase)/protein kinase B (AKT) pathway plays an important role in cancer cell proliferation, survival, motility, and metabolism signaling, and is one of the most frequently deregulated pathways in colon cancer [20]. PI3K/AKT activation is tightly controlled and dependent on extracellular growth signals and intracellular nutrients, such as glucose and amino acids [21] and many dietary compounds that inhibit the activation of PI3K/AKT have been proposed as potential anti-cancer preventives [22]. To investigate whether sorghum bran extracts possess inhibitory activity against the growth factor-induced PI3K/AKT signaling pathway, we pretreated human colon cancer cells with sorghum bran extracts (HP, SC and Sumac), followed by co-treated with IGF-1, using LY294002 (a selective inhibitor of PI3K) as a positive control. As a result, a marked induction of phospho-AKT was detected in the IGF-1 (insulin-like growth factor-1)-treated colon cancer cells. However, pretreatment with HP, SC, and Sumac suppressed IGF-1-stimulated phosphorylation of AKT in all colon cancer cells (Figure 4A–C).

### 2.5. High Phenolic Sorghum Bran Extracts Activate AMPK and Autophagy in Human Colon Cancer Cells

**Figure 5:**
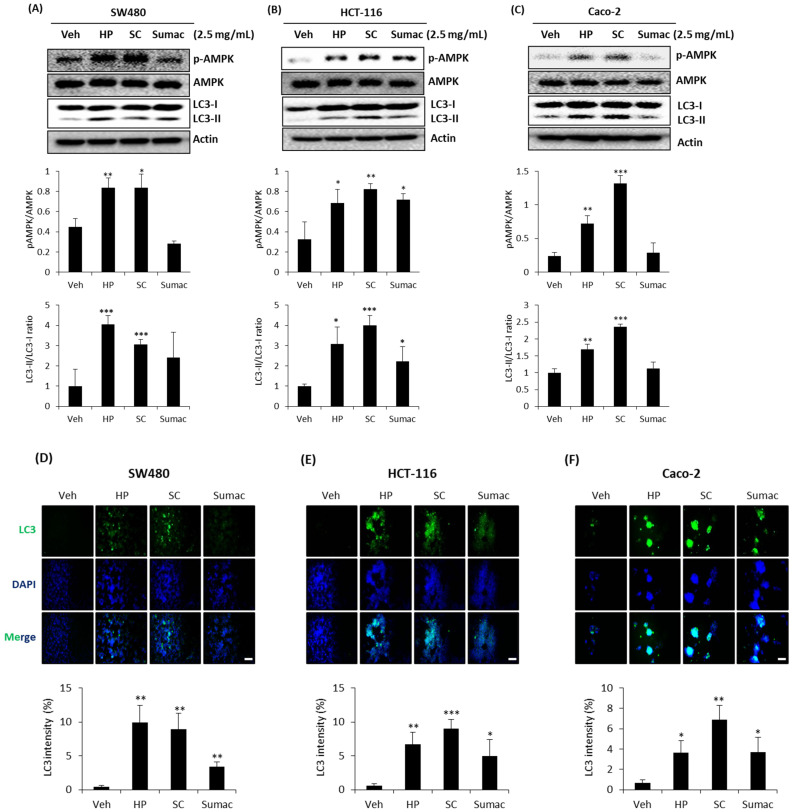
High phenolic sorghum bran extracts activate AMPK and autophagy in human colon cancer cells. (**A**–**C**) Three human colon cancer cells, SW480 (**A**), HCT-116 (**B**), and Caco-2 (**C**) cells, were treated with 2.5 mg/mL of sorghum bran extracts (HP, SC and Sumac) for 6 h and Western blotting was performed using antibodies against indicated proteins. (**D**–**F**) Immunofluorescence was performed to determine LC3 intensity in SW480 (**D**), HCT-116 (**E**) and Caco-2 (**F**) cells treated with 2.5 mg/mL of HP, SC and Sumac for 6 h. Scale bar represents 200 micrometers. Values are the mean ± SD (*n* = 3). Significant differences between the vehicle- and sorghum bran extract-treated groups are indicated as * *p* < 0.05, ** *p* < 0.01, *** *p* < 0.001.

AMP-activated protein kinase (AMPK) plays a significant role in maintaining cellular energy homeostasis and is activated in response to cellular energy levels. AMPK is emerging as a possible metabolic tumor suppressor and a target for cancer prevention and treatment [23]. Autophagy is a highly conserved process that targets proteins and damaged organelles for lysosomal degradation to maintain genomic integrity and cell survival, targeting autophagy as an attractive cancer therapeutic option [24]. Thus, natural polyphenolic compounds in the diet can trigger cancer cell death via various mechanisms through the canonical (Beclin-1 dependent) and non-canonical (Beclin-1 independent) routes of autophagy [25]. Since AMPK directly activates autophagy via the mammalian target of rapamycin (mTOR)-dependent and-independent pathways [26], we explored whether high phenolic sorghum extracts affect AMPK-mediated autophagy. AMPK activation, as measured by phosphorylation, was observed in all three cells treated with sorghum bran extracts, except for Sumac-treated SW480 and Caco-2 cells. Additionally, HP, SC, and Sumac activated the lysosomal turnover of the autophagosome marker LC3-II, reflecting the induction of autophagic activity in all three colon cancer cells, except Sumac-treated SW480 and Caco-2 cells (Figure 5A–C). Immunofluorescence data also showed an increased amount of LC3 in the cells treated with sorghum bran extracts (Figure 5D–F).

### 2.6. High Phenolic Sorghum Brans Repress the Formation of Colon Tumors in APC Min/+Mice

**Figure 6:**
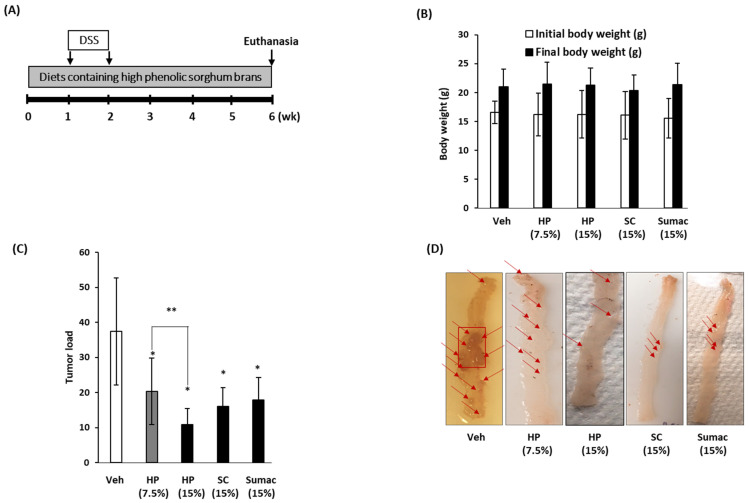

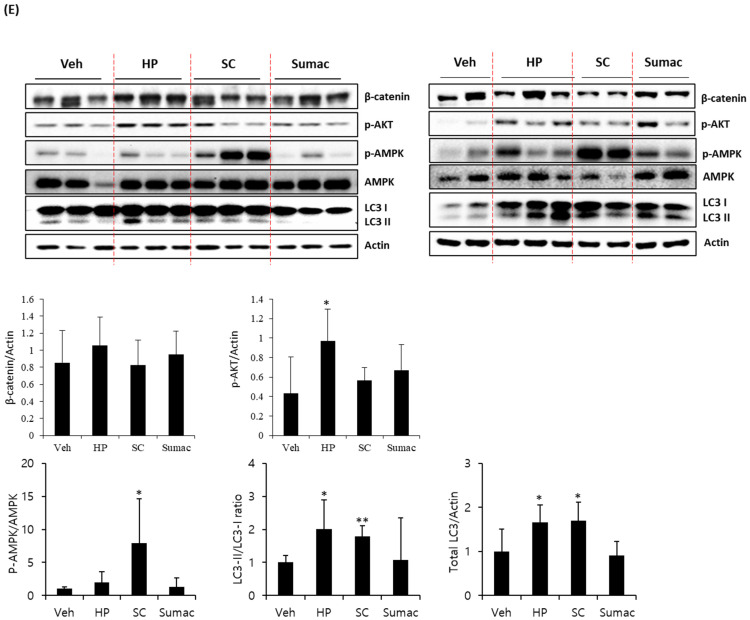
High phenolic sorghum brans repress the formation of colon tumors in *APC Min/+*mice. Total 53 *APC Min/+*mice were randomly assigned into one of five groups and fed either a control diet (*n* = 11) or diets containing low HP (7.5% *w/w*; *n* = 11), high HP (15% *w/w*; *n* = 11), SC (15%; *n* = 10) and Sumac (15%; *n* = 10) for 6 weeks. All groups were administered 2% DSS provided through drinking water. (**A**) Experimental design. (**B**) Initial and final body weight of experimental animals. (**C**) The tumor load is calculated based on the number and the size of tumors in colon and rectum. (**D**) Representative image of tumors. (**E**) Expression of indicated genes from colon tumor tissues obtained from vehicle (*n* = 5), 15% HP (*n* = 6), 15% SC (*n* = 5) and 15% Sumac (*n* = 5)-treated mice. Bar graphs indicate the average per group. Significant differences between the vehicle- and sorghum bran-treated groups are indicated as * *p* < 0.05, ** *p* < 0.01.

Finally, we investigated whether the consumption of high-phenolic sorghum bran-containing diets influences tumor formation using a DSS-treated APC Min/+colon cancer genetic mouse model. We prepared experimental diets containing low (7.5% *w/w*) and high doses (15% *w/w*) of HP and 15% *w/w* of SC and 15% *w/w* of Sumac (Table S1). APC Min/+ mice were fed the prepared diets for 6 weeks with 1 week of DSS treatment (Figure 6A). No differences in body weight were observed between the experimental mice at the end of the study and mice receiving a regular diet (Figure 6B). No toxicity was observed in any of the experimental groups of mice fed different types of sorghum bran. The number and size of colon tumors were expressed as the tumor load. As shown in Figure 6C,D, the mice fed HP (15% *w/w*), SC (15% *w/w*) and Sumac (15% *w/w*) showed significantly lower tumor loads (77%, 57% and 54%) compared to the control groups in the colon and rectum. A similar suppression of tumor formation was observed in the small intestine (data not shown). In addition, HP showed a dose-dependent inhibition of tumor formation; 43% inhibition in low (7.5% *w/w*) HP and 77% inhibition in high (15% *w/w*) HP. These data indicate that the addition of polyphenol-containing sorghum bran to the diet prevents tumor formation and progression. To determine whether treatment with sorghum bran affected the expression of associated genes, we performed Western blotting using colon tumor tissues isolated from each treatment group. As shown in Figure 6E, the expression of β-catenin was not altered by sorghum treatment, whereas an increase in p-AKT was observed only in the HP-treated group. Interestingly, supplementation with sorghum bran (HP and SC) led to an increase in total LC3 to LC3-II conversion, whereas a higher phosphorylation of AMPK was observed in SC-treated mice.

## 3. Discussion

There is a growing body of evidence that the increased consumption of whole grains containing phytochemicals is an effective way to prevent diverse types of cancer. Sorghum has received less attention due to the fact that the consumption of sorghum is relatively small compared to that of other cereals, despite showing beneficial action against chronic diseases in humans [27,28]. Since the most abundant bioactive component in sorghum is polyphenols, we screened several sorghum brans from the sorghum association panel and identified PI570481 as a novel high phenolic (HP) black sorghum genotype [12], and proposed optimal extraction conditions to maximize the extraction of phenolic compounds [13].

We previously observed the anti-cancer activity of HP, including the induction of growth arrest and apoptosis, and its ability to repress cancer cell invasion and migration [12,14]. A similar inhibition of cancer cell growth and activation of apoptosis was detected in cells treated with HP and SC (Figure 1) without change of viability in normal fibroblast cells (data not shown). In the current study, we focused on the cancer signaling pathways, including TNF-α-induced NF-κB and IGF-1-induced PI3K/AKT and AMPK pathways.

An interesting finding was the fact that specialty sorghum bran extracts (HP and SC) increased the transcriptional activity of NF-κB in SW480 and HCT-116 cells, but not in Caco-2 cells (Figure 2A–C). However, HP, SC, and Sumac inhibited TNF-α-induced NF-κB activity in HCT-116 cells (Figure 2B). These data suggest the dual activity of specialty sorghum bran extracts on NF-κB signaling in the presence and absence of its ligand, TNF-α. The dual activity of NF-κB in cancer cells has been reported previously [29,30]. We speculate that specialty sorghum bran extracts activate the DNA damage/NF-κB pathway to induce apoptosis in the absence of TNF-α, while inhibiting TNF-α/NF-κB-mediated cancer progression and invasion, because TNF-α is an important inflammatory factor and the TNF-α/NF-κB pathway contributes to tumor cell invasion and migration [31]. In addition, the downregulation of TNF-α-induced NF-κB was observed in HCT-116 cells (*APC* wild-type), but not in SW480 (*APC* mutant) and Caco-2 (*APC* mutant) cells. In the future, it will be important to determine whether the dual NF-κB activity of sorghum polyphenols is associated with crosstalk between APC and the NF-κB signaling pathway [32].

In the current study, we focused on β-catenin expression and transactivation since mutations in *APC* tumor suppressor genes are observed in 90% of colon cancer patients, and the end point of this mutation results in the activation of β-catenin transcriptional activity in human colon cancers. As shown in Figure 3, significant inhibitory activity was observed in SW480 and HCT-116 cells treated with HP and SC, while no significant change was detected in Caco-2 cells (Figure 3A–C), which was reflected in the expression of β-catenin (Figure 3D–F). We do not know exact reason for different potency of sorghum bran extracts depending on the cell types. We speculate that genetic alteration of key genes in these cells might be associated with different regulation of β-catenin signaling and different characteristics within the same adenocarcinoma. For example, unlike SW480 and HCT-116, Caco-2 cells are heterogenous subpopulations and composed of differing morphologies and possess the capacity of differentiation [33,34]. In addition, Sumac downregulated the transcriptional activity of β-catenin without changing β-catenin protein levels. This indicates that the Sumac-mediated repression of β-catenin transactivation is associated with other mechanism(s), such as protein–protein interactions (β-catenin and TCF4), rather than the transcription of β-catenin gene or stability of β-catenin protein.

Regarding the upstream regulator of β-catenin, we focused on PI3K/AKT, which is constitutively activated in many human colon cancers. As shown in Figure 4, pretreatment with high phenolic sorghum extracts weakened the IGF-1-stimulated phosphorylation of AKT in three human colon cancer cells, indicating that growth factor/receptors/PI3K/AKT pathways could be an efficient target for cancer prevention using high-phenolic sorghum bran.

Another interesting target signaling modulated by the specialty sorghum bran extract is AMPK-mediated autophagy (Figure 5). Overall, the activation of AMPK and subsequent autophagy, as measured by LC3 modification (conversion), was observed in cancer cells treated with HP and SC. Although Sumac increased the expression of total LC3 (Figure 5D–F), it did not affect the conversion of LC3 in two APC mutant cells (SW480 and Caco-2). Further studies are required to explore whether different responses in LC3 conversion by Sumac are associated with APC.

**Figure 7:**
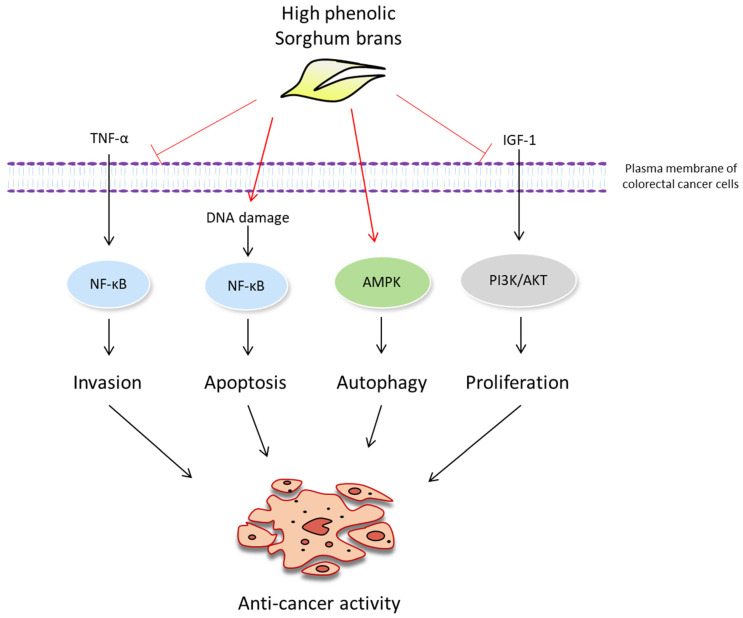
Proposed anti-cancer mechanisms of high phenolic sorghum brans.

Finally, to determine the physiological relevance of the in vitro findings, we tested and compared the feeding effect of HP, SC and Sumac sorghum bran using a colon cancer mouse model. Significant tumor suppressive activity of HP, SC and Sumac was observed in *APC Min/+* mice bearing benign polyp tumors. We also measured the expression of the target proteins tested in the in vitro studies. We observed an increased phosphorylation of AMPK and LC3-II expression, indicating that the activation of AMPK-dependent autophagy may be a common cancer preventive mechanism of sorghum bran, as observed in both invasive (SW480 and HCT-116) and non-invasive (Caco-2) cells (Figure 5). This is supported by the higher expression and conversion of LC3 from tissue samples (Figure 6E). However, we did not observe a difference in β-catenin levels in the tumor tissues. Since we observed a downregulation of β-catenin only in invasive (metastatic) SW480 and HCT-116 cells (Figure 3) and the tumor produced from *APC Min/+* mice is a benign adenoma, β-catenin may be a target of sorghum bran extracts only for invasive and metastatic cancer, but not in benign polyps. Figure 7 summarizes the proposed mechanisms responsible for the anti-cancer effect. One unexpected result of this study is that Sumac bran had comparable efficacy to sorghum brans much higher in polyphenols. There are several possible explanations for this: (1) after a certain level of polyphenols, adding more sorghum polyphenols does not increase the anti-cancer effect, (2) bran components other than polyphenols contribute to the anti-cancer effect of sorghum and (3) only unique polyphenols exert the anti-cancer effect. It will be important to incorporate low polyphenol white sorghum bran in future animal studies to confirm the role of sorghum polyphenols as an anti-cancer agent.

## 4. Materials and Methods

### 4.1. Materials

Human colon cancer cells (SW480, HCT-116 and Caco-2) were purchased from the American Type Culture Collection (Manassas, VA, USA). Antibodies against p-AKT (#4060), β-catenin (#9582), actin (#5125), p-AMPK (#2535), AMPK (#5831), and LC3 (#4108) were purchased from Cell Signaling Technology (Danvers, MA, USA). pNF-κB-Luc (#219078) was purchased from (Agilent Santa Clara, CA, USA). All chemicals, including cell culture media, were purchased from Fisher Scientific unless otherwise specified.

### 4.2. Sorghum Grain Processing

Black PI570481 (HP) is photo-sensitive, as described previously, and does not grow readily in the Midwest [13]. Brown PI534144/SC84 (SC) sorghum accessions are another sorghum genotype with high photo-insensitive polyphenols and readily grows in Midwestern states, such as Kansas [35]. Both HP and SC were grown in a winter nursery between December 2017 and March 2018 at Puerto Vallarta, Mexico. Commercially available sumac (referred to as “Sumac” throughout this manuscript) was purchased from NuLife Markets (Scott City, KS, USA). The bran was obtained by removing 15% of the grain through decortication, followed by milling to a particle size of 250 µM or less.

### 4.3. Preparation of High Phenolic Sorghum Bran Extract

Sorghum bran extracts were prepared using a previously described method [13]. In brief, 10% bran (*w/w*) was extracted using a solvent containing 70% ethanol and 5% citric acid at room temperature for 2 h, followed by storage at −20 °C overnight. Afterwards, the samples were centrifuged at 1000× *g* for 10 min and the supernatant was collected and used as a crude extract. The total phenolic content of the sorghum bran extracts was measured using the previously published Folin–Ciocalteu (FC) assay. [36] The total phenolic content of HP507481, SC84 and sumac bran extracts was measured to be 57.8, 30.3 and 17.6 mg gallic acid equivalents per 1 g of dry weight of sorghum bran (mg GAE/g), respectively. Although sumac sorghum contains lower phenolic content than PI570481 and SC84, it was used in this study because it contains a high phenolic content amongst grains commercially available for human consumption.

### 4.4. Cell Culture and Treatment

All human colon cancer cells (SW480, HCT-116, and Caco-2) were incubated at 37 °C in a humidified atmosphere of 5% CO_2_ using Dulbecco’s modified Eagle’s medium (DMEM) supplemented with 10% fetal bovine serum (FBS), 100 U/mL penicillin and 100 µg/mL streptomycin. High phenolic sorghum bran extract was diluted using 70% ethanol with 5% citric acid solvent (vehicle) into culture media to obtain final concentrations of 0, 1.25 and 2.50 mg/mL (*w/v*).

### 4.5. Cell Proliferation and Apoptosis

Cell proliferation was measured using the MTT assay, as described previously [37]. Briefly, the cells were plated in 96-well culture dishes overnight and then treated with different concentrations of sorghum bran extracts for 24 h. Then MTT was added to each well and 96-well culture dishes were incubated for 2 h at 37 °C. Culture media was removed, and the absorbance was measured in an ELISA plate reader (Bio-Tek Instruments, Winooski, VT, USA) after 100 μL of dimethyl sulfoxide was added. Apoptosis was measured using the Caspase-Glo 3/7 assay kit (Promega, Madison, WI, USA), as previously described [38]. Briefly, the cells were plated in 96-well culture dishes overnight and then treated with different concentrations of sorghum bran extracts for 24 h. In total, 50 μL of Caspase-Glo 3/7 assay solution was directly added into each well and the luminescence was measured after incubation at 37 °C for 1 h.

### 4.6. Western Blotting

Western blotting was performed as previously described [37]. In brief, cell lysates were collected by lysing the cells in radioimmunoprecipitation assay buffer supplemented with protease and phosphatase inhibitor cocktail and centrifuged at 12,000× *g* for 10 min at 4 °C. After determination of protein concentration, equal amounts of proteins were subjected to SDS-PAGE and transferred onto nitrocellulose membranes. The membranes were blocked for non-specific binding with 5% non-fat milk in Tris-buffered saline containing 0.05% Tween 20 for 1 h at room temperature and then probed with primary antibodies overnight at 4 °C. The next day, the membrane was incubated with horse radish peroxidase-conjugated immunoglobulin G for 1 h at room temperature. Chemiluminescence was detected with Pierce ECL Western Blotting substrate (Thermo Scientific, Waltham, MA, USA) and visualized by Chemidoc MP Imaging system (Bio-Rad, Hercules, CA, USA).

### 4.7. Immunofluorescence

The cells were serum-starved overnight and treated with sorghum extracts for 6 h. Cells were washed twice with PBS and fixed with 10% formalin for 10 min. After washing, the cells were incubated with 0.2% Triton X-100 for permeabilization for 10 min and blocked with 1% BSA for 1 h. Then, the cells were incubated with LC3 antibody (1:200) at 4 °C overnight. After washing, the cells were incubated with a secondary antibody (Invitrogen, #A21206, Waltham, MA, USA) for 2 h at room temperature. Finally, the cells were mounted with mounting medium containing DAPI (#sc-24941; Santa Cruz, CA, USA) and observed under a fluorescence microscope (ECLIPS Ti; Nikon, Melville, NY, USA). Fluorescence intensity was determined using ImageJ software (https://imagej.nih.gov/ij/ (accessed on 1 February 2021)).

### 4.8. Transient Transfection and Luciferase Assay

To determine the transcriptional activity of NF-κB, the luciferase reporter gene (pNF-κB-Luc; 1 µg) including NF-κB binding sites was co-transfected with *pRL-null* (0.1 µg) using Lipofectamine 3000 (Thermo Fisher Scientific, Waltham, MA, USA) to the cells for 24 h. Then, the transfected cells were pre-incubated with the media containing 0 and 2.5 mg/mL of sorghum bran extracts for 1 h and co-treated with TNF-α (10 ng/mL) for 6 h. For the transcriptional activity of β-catenin, the TOP/FOP flash reporter gene plasmids (1 µg) [39] were co-transfected with *pRL-null* (0.1 µg) using Lipofectamine 3000 (Thermo Fisher Scientific, Waltham, MA, USA) or a Polyjet DNA transfection reagent (SignaGen Laboratories, Ijamsville, MD, USA) for 48 h. The transfected cells were then incubated with media containing 0, 1.25, and 2.5 mg/mL of sorghum bran extracts for 24 h. After the extraction of the cell lysates, the luciferase activity was measured using a dual-luciferase assay system (Promega, Madison, WI, USA) according to the manufacturer’s instructions.

### 4.9. Formulation of Mouse Diets

Total moisture, protein, crude fat, fiber, crude fiber, ash, and carbohydrates were measured by Eurofins Scientific Inc. (Des Moines, IA, USA). The diets were matched for the total amount of calories, as well as fat, carbohydrate, protein, and fiber content, in order to remove any artifacts resulting from differences in macronutrients. The pellets were formulated based on the AIN-76A diet (control), without heating (Research Diets Inc. New Brunswick, NJ, USA) (Supplementary Table S1).

### 4.10. In Vivo Study

*APC Min/+ mice* were purchased from the Jackson Laboratory (Bar Harbor, ME, USA), bred, and genotyped as described previously [40]. Genotyping was performed using three PCR primer sets (5′-TTCTGAGAAAGACAGAAGTTA-3′ 5′-TTCCACTTTGGCATAAGGC-3′ 5′-GCCATCCCTTCACGTTAG-3′) as previously described [40]. A total of 53 *APC Min/+* mice (aged 4–6 weeks) were assigned to one of the five groups and fed with different diets, including diets containing no sorghum bran (control; *n* = 11, 8 female + 3 male), low dose of HP bran (7.5% *w/w*; *n* = 11, 7 female + 4 male) and high doses of HP (15% *w/w*; *n* = 11, 8 female + 3 male), SC (15% *w/w*; *n* = 10, 7 female + 3 male), and Sumac (15% *w/w*; *n* = 10, 7 female + 3 male) sorghum bran for 6 weeks (Figure 6A). Diets containing different sorghum brans were provided continuously for 6 weeks and 2% DSS was provided to all groups through drinking water for 1 week. After 6 weeks on the diet (4 weeks after discontinuing 2% DSS treatment), all mice were euthanized using carbon dioxide asphyxiation. Mouse colons and rectums were removed, washed, and cut open longitudinally. The number and size of polyps were measured and calculated as the tumor load, as described previously [40]. The animal study was approved by the Institutional Animal Care and Use Committee (IACUC) of the University of Maryland.

### 4.11. Statistical Analysis

Data are expressed as the mean ± standard deviation (SD), as indicated in the figure legends. Statistical analysis was performed using Student’s *t*-test, where differences were considered significant at *p* < 0.05.

## Data Availability

All data is published within the manuscript. Original images and data can be obtained by contacting one of the corresponding authors.

## Supplementary Materials

The following are available online at https://www.mdpi.com/article/10.3390/ijms22158286/s1.

## References

[1] Kothari K., Ale S., Bordovsky J.P., Munster C.L. (2020). Assessing the Climate Change Impacts on Grain Sorghum Yield and Irrigation Water Use under Full and Deficit Irrigation Strategies. Trans. ASABE.

[2] Aruna C., Visarada K., Bhat B.V., Tonapi V.A. (2019). Chapter 1—Sorghum: A Bundle of Opportunities in the 21st Century. Breeding Sorghum for Diverse End Uses.

[3] Ratnavathi C.V. (2019). Chapter 12—Grain Structure, Quality, and Nutrition. Breeding Sorghum for Diverse End Uses.

[4] Wu G., Bennett S.J., Bornman J.F., Clarke M., Fang Z., Johnson S.K. (2017). Phenolic profile and content of sorghum grains under different irrigation managements. Food Res. Int..

[5] Zhang Y., Li M., Gao H., Wang B., Tongcheng X., Gao B., Yu L. (2019). Triacylglycerol, fatty acid, and phytochemical profiles in a new red sorghum variety (Ji Liang No. 1) and its antioxidant and anti-inflammatory properties. Food Sci. Nutr..

[6] Li M., Xu T., Zheng W., Gao B., Zhu H., Xu R., Deng H., Wang B., Wu Y., Sun X. (2021). Triacylglycerols compositions, soluble and bound phenolics of red sorghums, and their radical scavenging and anti-inflammatory activities. Food Chem..

[7] Yang L., Browning J.D., Awika J.M. (2009). Sorghum 3-deoxyanthocyanins possess strong phase II enzyme inducer activity and cancer cell growth inhibition properties. J. Agric. Food Chem..

[8] Ritchie L.E., Sturino J.M., Carroll R.J., Rooney L.W., Azcarate-Peril M.A., Turner N.D. (2015). Polyphenol-rich sorghum brans alter colon microbiota and impact species diversity and species richness after multiple bouts of dextran sodium sulfate-induced colitis. FEMS Microbiol. Ecol..

[9] Ritchie L.E., Taddeo S.S., Weeks B.R., Carroll R.J., Dykes L., Rooney L.W., Turner N.D. (2017). Impact of Novel Sorghum Bran Diets on DSS-Induced Colitis. Nutrients.

[10] Turner N., Diaz A., Taddeo S., Vanamala J., McDonough C., Dykes L., Murphy M., Carroll R., Rooney L. (2006). Bran from black or brown sorghum suppresses colon carcinogenesis (ABSTRACT). FASEB J..

[11] Park J.H., Darvin P., Lim E.J., Joung Y.H., Hong D.Y., Park E.U., Park S.H., Choi S.K., Moon E.-S., Cho B.W. (2012). Hwanggeumchal sorghum induces cell cycle arrest, and suppresses tumor growth and metastasis through Jak2/STAT pathways in breast cancer xenografts. PLoS ONE.

[12] Smolensky D., Rhodes D., McVey D.S., Fawver Z., Perumal R., Herald T., Noronha L. (2018). High-Polyphenol Sorghum Bran Extract Inhibits Cancer Cell Growth Through ROS Induction, Cell Cycle Arrest, and Apoptosis. J. Med. Food.

[13] Cox S., Noronha L., Herald T., Bean S., Lee S.-H., Perumal R., Wang W., Smolensky D. (2019). Evaluation of ethanol-based extraction conditions of sorghum bran bioactive compounds with downstream anti-proliferative properties in human cancer cells. Heliyon.

[14] Lee S.-H., Lee J., Herald T., Cox S., Noronha L., Perumal R., Lee H.-S., Smolensky D. (2020). Anticancer Activity of a Novel High Phenolic Sorghum Bran in Human Colon Cancer Cells. Oxidative Med. Cell. Longev..

[15] Awika J.M., Rooney L.W. (2004). Sorghum phytochemicals and their potential impact on human health. Phytochemistry.

[16] Rhodes D., Gadgil P., Perumal R., Tesso T., Herald T.J. (2017). Natural Variation and Genome-Wide Association Study of Antioxidants in a Diverse Sorghum Collection. Cereal Chem..

[17] Uchimiya M. (2020). Proton-Coupled Electron Transfers of Defense Phytochemicals in Sorghum (*Sorghum bicolor* (L.) Moench). J. Agric. Food Chem..

[18] Soleimani A., Rahmani F., Ferns G.A., Ryzhikov M., Avan A., Hassanian S.M. (2020). Role of the NF-kappaB signaling pathway in the pathogenesis of colorectal cancer. Gene.

[19] Yu W.-K., Xu Z.Y., Yuan L., Mo S., Xu B., Cheng X.-D., Qin J.-J. (2020). Targeting beta-Catenin Signaling by Natural Products for Cancer Prevention and Therapy. Front. Pharmacol..

[20] Zhang J., Roberts T.M., Shivdasani R.A. (2011). Targeting PI3K signaling as a therapeutic approach for colorectal cancer. Gastroenterology.

[21] Danielsen S.A., Eide P.W., Nesbakken A., Guren T., Leithe E., Lothe R.A. (2015). Portrait of the PI3K/AKT pathway in colorectal cancer. Biochim. Biophys. Acta.

[22] Suvarna V., Murahari M., Khan T., Chaubey P., Sangave P. (2017). Phytochemicals and PI3K Inhibitors in Cancer-An Insight. Front. Pharmacol..

[23] Li W., Saud S.M., Young M.R., Chen G., Hua B. (2015). Targeting AMPK for cancer prevention and treatment. Oncotarget.

[24] Codina N.S., Mancias J.D., Kimmelman A.C. (2017). The Role of Autophagy in Cancer. Annu. Rev. Cancer Biol..

[25] Hasima N., Ozpolat B. (2014). Regulation of autophagy by polyphenolic compounds as a potential therapeutic strategy for cancer. Cell Death Disease.

[26] Kim J., Kundu M., Viollet B., Guan K.-L. (2011). AMPK and mTOR regulate autophagy through direct phosphorylation of Ulk1. Nat. Cell Biol..

[27] Amarakoon D., Lou Z., Lee W., Smolensky D., Lee S. (2020). A mechanistic review: Potential chronic disease-preventive properties of sorghum. J. Sci. Food Agric..

[28] de Morais Cardoso L., Pinheiro S.S., Martino H.S., Pinheiro-Sant’Ana H.M. (2017). Sorghum (*Sorghum bicolor* L.): Nutrients, bioactive compounds, and potential impact on human health. Crit. Rev. Food Sci. Nutr..

[29] Perkins N.D. (2004). NF-kappaB: Tumor promoter or suppressor?. Trends Cell Biol..

[30] Hoesel B., Schmid J.A. (2013). The complexity of NF-kappaB signaling in inflammation and cancer. Mol. Cancer.

[31] Wu Y., Zhou B.P. (2010). TNF-alpha/NF-kappaB/Snail pathway in cancer cell migration and invasion. Br. J. Cancer.

[32] Deng J., Xia W., Miller S.A., Wen Y., Wang H.Y., Hung M.C. (2004). Crossregulation of NF-kappaB by the APC/GSK-3beta/beta-catenin pathway. Mol. Carcinog..

[33] Sambuy Y., De Angelis I., Ranaldi G., Scarino M.L., Stammati A., Zucco F. (2005). The Caco-2 cell line as a model of the intestinal barrier: Influence of cell and culture-related factors on Caco-2 cell functional characteristics. Cell Biol. Toxicol..

[34] Walter E., Kissel T. (1995). Heterogeneity in the human intestinal cell line Caco-2 leads to differences in transepithelial transport. Eur. J. Pharm. Sci..

[35] Rhodes D.H., Hoffmann L., Rooney W.L., Ramu P., Morris G.P., Kresovich S. (2014). Genome-wide association study of grain polyphenol concentrations in global sorghum [*Sorghum bicolor* (L.) Moench] germplasm. J. Agric. Food Chem..

[36] Herald T.J., Gadgil P., Tilley M. (2012). High-throughput micro plate assays for screening flavonoid content and DPPH-scavenging activity in sorghum bran and flour. J. Sci. Food Agric..

[37] Clark R., Lee J., Lee S.H. (2015). Synergistic anticancer activity of capsaicin and 3,3′-diindolylmethane in human colorectal cancer. J. Agric. Food Chem..

[38] Lee S.-H., Bahn J.H., Whitlock N.C., Baek S.J. (2010). Activating transcription factor 2 (ATF2) controls tolfenamic acid-induced ATF3 expression via MAP kinase pathways. Oncogene.

[39] Lee S.-H., Richardson R.L., Dashwood R.H., Baek S.J. (2012). Capsaicin represses transcriptional activity of beta-catenin in human colorectal cancer cells. J. Nutr. Biochem..

[40] Baek S., Okazaki R., Lee S.-H., Martinez J., Kim J.-S., Yamaguchi K., Mishina Y., Martin D.W., Shoieb A., McEntee M. (2006). Nonsteroidal anti-inflammatory drug-activated gene-1 over expression in transgenic mice suppresses intestinal neoplasia. Gastroenterology.

