# Supplementary material for: Polyphenol Containing Sorghum Brans Exhibit an Anti-Cancer Effect in Apc Min/+ Mice Treated with Dextran Sodium Sulfate
Source: Int J Mol Sci. 2021 Aug 1;22(15):8286. doi: 10.3390/ijms22158286 (PMC8347436; doi:10.3390/ijms22158286)
Supplement: Supplementary file 1 [file ijms-22-08286-s001.zip › ijms-1292493-supplementary.pdf]

Supplementary Table S1. Formulation of mouse diets

		15% HP	7.5% HP	15% SC	15% Sumac
**Ingredient**	gm	gm	gm	gm	gm
**Casein**	200	176.7	188.3	183.2	178.5
**DL-Methionine**	3	3	3	3	3
**Corn Starch**	150	44.5	97.1	40.5	44.2
**Sucrose**	500	500	500	500	500
**Percent of total carbohydrates from sorghum bran**	0 %	16.2%	8.13%	16.8%	16.3%
**Cellulose**	50	35.6	42.8	36.8	43.7
**High Phenolic (HP) Sorghum Bran**	0	150	75.2	0	0
**SC84 (SC) Sorghum Bran**	0	0	0	150	0
**Sumac Sorghum Bran**	0	0	0	0	150.8
**Corn Oil**	50	42.5	46.2	41.3	40.2
**Mineral Mix S10001**	35	35	35	35	35
**Vitamin Mix V10001**	10	10	10	10	10
**Choline Bitartrate**	2	2	2	2	2
**FD&C Yellow Dye #5**	0	0	0	0.025	0.05
**FD&C Red Dye #40**	0	0.05	0.025	0.025	0
**FD&C Blue Dye #1**	0	0	0.025	0	0

**Total**	1000	999.35	999.65	1001.85	1007.45
**High Phenolic Sorghum Bran (gm%)**	0%	15.00%	7.50%	0%	0%
**SC Sorghum Bran (gm%)**	0%	0%	0%	15.00%	0%
**Sumac Sorghum Bran (gm%)**	0%	0%	0%	0%	15.00%
**Protein**	177	177	177	177	177
**Carbohydrate**	660	660	660	660	660
**Sugar**	510	510	510	510	510
**Fat**	50	50	50	50	50
**Fiber**	50	50	50	50	50
**Protein**	17.7	17.7	17.7	17.7	17.6
**Carbohydrate**	66	66	66	65.9	65.5
**Fat**	5	5	5	5	5
**Fiber**	5	5	5	5	5
**Protein**	*708*	*708*	*708*	*708*	*708*
**Carbohydrate**	*2640*	*2640*	*2640*	*2640*	*2640*
**Fat**	*450*	*450*	*450*	*450*	*450*
**Total**	*3798*	*3798*	*3798*	*3798*	*3798*
**Protein**	*19*	*19*	*19*	*19*	*19*
**Carbohydrate**	*70*	*70*	*70*	*70*	*70*
**Fat**	*12*	*12*	*12*	*12*	*12*
**kcal / gm**	3.8	3.8	3.8	3.79	3.77

gm = gram
